# Evaluation of Resistance in Soybean Varieties to *Rotylenchulus reniformis* in Louisiana Fields

**DOI:** 10.2478/jofnem-2025-0039

**Published:** 2025-12-07

**Authors:** Lucy Kiarie, Paul P. Price, Tristan T. Watson

**Affiliations:** LSU AgCenter, Department of Plant Pathology and Crop Physiology, Baton Rouge 70803, LA; LSU AgCenter, Macon Ridge Research Station, Winnsboro 71295, LA

**Keywords:** *Glycine max* (L.) Merrill, resistance, host susceptibility, Louisiana fields, reniform nematode, *Rotylenchulus reniformis*, soybean

## Abstract

The reniform nematode (*Rotylenchulus reniformis*) is one of the most problematic nematodes affecting soybean (*Glycine max* (L.) Merrill) in Louisiana. Although reniform nematode-resistant soybean varieties have been identified in previous studies, the response of soybean varieties grown in Louisiana remains unclear. This study evaluated reniform nematode population development and yield using a series of field trials. It focused on commonly planted soybean varieties, as well as University of Missouri varieties with reported resistance to the reniform nematode. Field trials were conducted in 2022 and 2023 at two locations in Louisiana. The resistant soybean varieties consistently suppressed reniform nematode soil population densities, achieving population reductions of 73% in 2022 and 72% in 2023, relative to the commercially planted soybean varieties. Resistant soybean varieties generally achieved higher yields than the commercial soybean varieties. This trend was consistent across the field trials conducted over both years. Overall, this study has demonstrated that resistant soybean varieties can be an effective tool in reducing reniform nematode populations in infested fields and often achieve higher yields compared to the currently grown soybean varieties in Louisiana.

Soybean (*Glycine max* (L.) Merrill) is among the most economically important crops globally (FAO, 2023). The United States ranks among the top 10 soybean-producing countries globally. Several states, including Louisiana, contribute substantially to the overall soybean output (USDA/NASS, 2024a, 2024b). Soybean plays a significant part in the region's agricultural industry. However, numerous soybean production challenges, including extreme weather events, insect pests, diseases, and plant-parasitic nematodes, pose a significant threat to production (Roth et al., 2020).

The reniform nematode (*Rotylenchulus reniformis*) is the major problematic species within the genus *Rotylenchulus* in the United States (Robinson et al., 1997; Robinson, 2008). It was first observed in the southern United States in 1940 in a cotton field in Randolph County, Georgia, and was reported in Louisiana cotton production by 1941 (Smith and Taylor, 1941). Since its discovery, reniform nematode has become one of the most economically important and predominant plant-parasitic nematodes throughout the southern soybean producing states, including Louisiana (Overstreet and McGawley, 1998; Allen et al., 2021). Its ability to persist in soil for multiple years and high reproduction rate have contributed to the reniform nematode becoming a persistent threat to soybean, cotton, and sweetpotato in the region (Robbins et al., 1994b; Westphal and Smart, 2003; Koenning et al., 2004; Faske et al., 2024). Various management strategies for reniform nematode have been proposed to maintain nematode densities in soil below the economic threshold. In Louisiana, soybean fields are generally at risk of damage when soil population densities of reniform nematode exceed 2,000 nematodes per 500 cm^3^ of soil. Proposed management practices in Louisiana include the use of nematicides, crop rotation with a non-host, such as corn or grain sorghum, and the use of resistant varieties (Overstreet et al., 2014; Overstreet, 2015). When available, the use of resistant varieties is often the preferred management option to suppress nematode population density in soil, limit yield losses, and maintain economic profitability (Westphal and Scott, 2005). Some soybean varieties, which are resistant to reniform nematode, have been identified in previous studies, including ‘Forrest’, ‘Hartwig’, Pioneer 94B45, UAP Mid-South Dyna-Gro 3682, and Deltapine Seed DP 7375 RR (Robbins et al., 2001, 2002). In these studies, the varieties ‘Forrest’ and ‘Hartwig’ have been used as standard checks in resistance screening trials. The susceptibility of commercially planted soybean varieties to reniform nematode is largely unknown in Louisiana; however, preliminary field trials utilizing 21 commonly planted commercial varieties demonstrated high susceptibility among all varieties tested (Watson and Price, 2022). New reniform nematode-resistant soybean varieties have been developed by the University of Missouri (Chen et al., 2023, 2024a, 2024b); however, the utility of these new varieties to reduce reniform nematode population development in a field setting is also unknown. This study aimed to evaluate the reniform nematode population development on locally planted soybean varieties and new varieties reported to have resistance, as well as their corresponding yield.

## Materials and Methods

### Site descriptions

The first field was at the LSU AgCenter Northeast Research Station (NERS) in St. Joseph, Louisiana, United States (31.943014, −91.226881). The soil in the site is bruin silt loam (49% sand, 42% silt, 9% clay; 0.8% organic matter; 5.8 pH; 4.8 meq/100 g cation exchange capacity). The NERS field has a history of severe infestation with reniform nematode and moderate infestation with southern root-knot nematode (*Meloidogyne incognita*). The second field was located at the LSU AgCenter Macon Ridge Research Station (MRRS) in Winnsboro, Louisiana, United States (32.137166, −91.697141). The site's soil is gigger silt loam (35% sand, 48% silt, 17% clay; 1.3% organic matter; 5.3 pH; 6.3 meq/100 g cation exchange capacity). The MRRS field has a history of severe infestation with the reniform nematode. Both fields were planted with cotton the year before planting the soybean trials and followed Louisiana's commercial production practices concerning the application of fungicides, insecticides, fertilization, and irrigation.

### Experimental design and measurements

In both NERS and MRRS fields, the experimental design was a randomized complete block design with five replicate plots of each variety. Top-selling soybean varieties commonly grown in Louisiana were selected for evaluation. In 2022, 12 soybean varieties were evaluated, whereas in 2023, only 10 soybean varieties were included in the experiment due to seed availability (Table 1). In 2022, the trials were planted on 17 May 2022 at MRRS and 19 May 2022 at NERS. In 2023, the trials were planted on 18 May 2023 at NERS and 23 May 2023 at MRRS in a separate portion of the field from the previous year. At the NERS field, plots were four rows wide on 96.5 cm row spacing and were 10.7 m long. At the MRRS, plots were four rows wide on 101.6 cm row spacing and were 10.7 m long. The soybean seeding rate at both fields was 120,000 seeds/ha. In the 2022 field trial, AgLogic 15GG (active ingredient, aldicarb) was included in the experiment as a sub-plot treatment to evaluate the performance of soybean varieties with and without the presence of nematode pressure. The nematicide was applied in-furrow to two of the four treatment rows as granules at a rate of 16.8 kg/ha at the time of planting.

**Table 1:** Soybean varieties evaluated in two *Rotylenchulus reniformis*-infested fields in Louisiana in 2022 and 2023.

**Soybean varieties**	
**2022**	**2023**
Bayer AG48XF2	Bayer AG48XF0
Dyna-Gro S48XT40	Bayer AG48XF2
Pioneer P46A86X	Bayer AG48X9
Progeny P4444RXS	Great Heart Seed GT-4677XS
Progeny P5252RX	Local Seed Company LS5009XS
University of Missouri S16-5503GT	Pioneer P54A54X
University of Missouri S16-5540R	University of Missouri S16-5503GT
University of Missouri S16-16814R	University of Missouri S16-5540R
University of Missouri S16-16641R	University of Missouri S16-16814R
University of Missouri S11-20195R	University of Missouri S16-16641R
WinField United Seed 48-D03	
WinField United Seed 46-D09	

Stand count (number of plants per hectare) was measured in each subplot at 14 and 28 days after planting (DAP) for each field. Four subsections measuring 1.52 m were randomly selected during each measurement, and the number of plants within a row was counted in each subsection. Reniform nematode population densities in soil were evaluated at planting, mid-season (56 DAP), and harvest in sub-plots from each field. Twelve soil cores at a depth of 20–25 cm and 2.5 cm in diameter were collected randomly from each sub-plot during each sampling date. During the mid-season and at harvest soil sampling, the soil cores were obtained next to the crop stem (≈5 cm). The soil cores from each plot were mixed in a plastic bucket and stored in a plastic bag at 12°C for approximately 14 days before processing. The nematodes were extracted from a 250 cm^3^ soil sub-sample using a semi-automated elutriator and sucrose centrifugal flotation method (Jenkins, 1964; Byrd et al., 1976). Nematodes were collected over a 25 μm sieve. The nematodes recovered were transferred in water into 20 mL plastic vials and stored at 4°C before quantification under a compound microscope. The data are presented as the number of nematodes per 500 cm^3^ of soil. At the end of the growing season, soybeans were harvested with a two-row combine when the seed moisture was approximately 13% in both fields for all years (2022, 2023). Yield measurements for each field were taken and recorded in kilograms per hectare corrected to a moisture content of 13%.

### Statistical analysis

Data analysis and fitting of the models were performed using a written R script run in the RStudio software version 4.3.2 (R Foundation for Statistical Computing Platform). In 2022, data from each field were analyzed using a two-way analysis of variance (ANOVA) with soybean variety and nematicide as factors. When there was no interaction effect among factors, each factor was analyzed separately as a one-way ANOVA. In 2023 (no nematicide sub-plot treatment), a one-way ANOVA test was run to analyze the data for each field. Non-parametric tests, Mann–Whitney *U*-test and Kruskal–Wallis test, were performed to analyze *M. incognita* data for 2022 and 2023, respectively. The Fisher's least significant difference (LSD) test was used to examine treatment means differences (*P* < 0.05).

## Results

### 2022 soybean growing season

At the NERS field in 2022, the reniform nematode soil population densities averaged 2,048–4,843 nematodes per 500 cm^3^ of soil at the time of planting (Table 2). By mid-season, differences in reniform nematode soil population densities were observed among soybean varieties tested. Field plots planted with the resistant University of Missouri soybean varieties showed reduced reniform nematode soil population densities during the mid-season sampling relative to field plots planted with the other commercially available soybean varieties. No differences in reniform nematode soil population densities were observed between plots treated with AgLogic 15GG and non-treated plots at mid-season. At harvest, differences in final reniform nematode soil population densities were observed. The field plots planted with the resistant University of Missouri soybean varieties continued to maintain lower reniform nematode soil population densities, resulting in a 79% nematode reduction relative to field plots planted with other soybean varieties. The reniform nematode soil population densities did not differ between field plots treated with AgLogic 15GG and non-treated plots at the end of the growing season. The southern root-knot nematode was undetectable in field plots at the time of planting. By mid-season, the southern root-knot nematode was detectable; however, no differences in root-knot nematode soil population densities were observed among the varieties or nematicide treatments. Similarly, at harvest, no differences in southern root-knot nematode soil population densities were observed among the tested varieties or nematicide treatments. Differences in stand count were observed among certain varieties at each assessment date (Table 3). At 14 DAP, Bayer AG48XF2, Dyna-Gro S48XT40, Pioneer P46A86X, Progeny P5252RX, University of Missouri S16-16641R, and WinField United Seed 46-D09 had significantly more plants per hectare than Progeny P4444RXS, University of Missouri S16-5503GT, University of Missouri S16-5540R, University of Missouri S16-16814R, and University of Missouri S11-2019R. At 28 DAP, Bayer AG48XF2, Pioneer P46A86X, and University of Missouri S16-16641R continued to maintain the greatest density of plants per hectare, whereas Progeny P444RXS, University of Missouri S16-5503GT, and University of Missouri S11-20195R had significantly fewer plants per hectare. Nematicide application did not influence plant establishment at either measurement date. Differences in yield were observed among tested varieties, with Pioneer P46A86X, University of Missouri S16-5503GT, University of Missouri S16-5540R, and University of Missouri S11-20195R providing significantly higher yields than Progeny P4444RXS, Progeny P5252RX, and University of Missouri S16-16641R. Nematicide application did not affect the yield of the tested varieties.

**Table 2:** Influence of soybean variety and nematicide on *Rotylenchulus reniformis* and *Meloidogyne incognita* soil population densities throughout the growing season at the NERS field in 2022.

**Factor**	**Level**	***R. reniformis*/500 cm^3^ soil**			***M. incognita*/500 cm^3^ soil**		

		**At plant**	**Mid-season**	**At harvest**	**At plant**	**Mid-season**	**At harvest**
Variety	Bayer AG48XF2	3,018 a	19,712 ab	3,352 ab	0	80 a	200 a
	Dyna-Gro S48XT40	4,843 a	21,474 a	3,376 ab	0	0 a	88 a
	Pioneer P46A86X	3,392 a	19,512 ab	4,296 a	0	0 a	96 a
	Progeny P4444RXS	4,008 a	13,424 b	2,808 b	0	16 a	136 a
	Progeny P5252RX	2,912 a	20,320 ab	2,204 bc	0	112 a	72 a
	University of Missouri S16-5503GT	4,080 a	3,792 c	512 d	0	0 a	112 a
	University of Missouri S16-5540R	3,991 a	2,864 c	560 d	0	0 a	192 a
	University of Missouri S16-16814R	2,784 a	1,904 c	568 d	0	0 a	136 a
	University of Missouri S16-16641R	3,167 a	2,160 c	736 d	0	32 a	256 a
	University of Missouri S11-20195R	3,516 a	3,072 c	1,056 cd	0	48 a	256 a
	WinField United Seed 48-D03	2,612 a	17,498 ab	3,296 ab	0	0 a	104 a
	WinField United Seed 46-D09	2,048 a	16,064 ab	3,040 ab	0	0 a	120 a

Nematicide	None	3,525 a	10,620 a	2,246 a	0	29 a	175 a
	AgLogic 15GG	3,203 a	13,013 a	2,055 a	0	19 a	120 a

*P*-values	Variety	0.595	<0.001	<0.001	-	0.274	0.583
	Nematicide	0.507	0.117	0.509	-	0.974	0.070
	Interaction	0.992	0.534	0.184	-	0.250	0.904

For each factor, values with common letters within a column do not differ significantly (*P*-value >0.05).

NERS, Northeast Research Station.

**Table 3:** Influence of soybean variety on stand count and yield at the NERS field in 2022.

**Factor**	**Level**	**Stand count (plants/ha)**		
		**14 DAP**	**28 DAP**	**Yield (kg/ha)**
Variety	Bayer AG48XF2	318,837 a	287,225 a	3,968 b–d
	Dyna-Gro S48XT40	318,158 a	235,560 cd	4,035 b–d
	Pioneer P46A86X	311,021 ab	282,127 ab	4,237 ab
	Progeny P4444RXS	262,072 c	215,505 d	3,833 d
	Progeny P5252RX	305,582 ab	247,797 b–d	3,766 d
	University of Missouri S16-5503GT	207,346 d	168,597 e	4,506 a
	University of Missouri S16-5540R	269,211 c	234,199 cd	4,371 a
	University of Missouri S16-16814R	268,870 c	239,978 cd	4,035 b–d
	University of Missouri S16-16641R	311,360 ab	256,295 a–c	3,766 d
	University of Missouri S11-20195R	173,354 e	143,103 e	4,170 a–c
	WinField United Seed 48-D03	285,186 bc	242,697 cd	4,035 b–d
	WinField United Seed 46-D09	309,660 ab	224,683 cd	3,901 cd

Nematicide	None	279,577 a	233,519 a	4,035 a
	AgLogic 15GG	277,200 a	229,439 a	4,035 a

*P*-values	Variety	<0.001	<0.001	<0.001
	Nematicide	0.682	0.576	0.984
	Interaction	0.079	0.999	0.608

For each factor, values with common letters within a column do not differ significantly (*P*-value >0.05).

DAP, days after planting; NERS, Northeast Research Station.

At the MRRS field in 2022, reniform nematode soil population densities averaged 17,624–41,457 nematodes per 500 cm^3^ of soil at the time of planting (Table 4). By mid-season, differences in reniform nematode soil population densities were observed among the varieties tested. In the field plots planted with the resistant University of Missouri soybean varieties, reniform nematode soil population densities declined relative to the field plots planted with other soybean varieties. No differences were observed in reniform nematode soil population densities between the plots treated with AgLogic 15GG and non-treated plots at mid-season sampling. At harvest, differences in reniform nematode soil population densities were observed among the tested varieties. The field plots planted with the resistant University of Missouri soybean varieties continued to suppress reniform nematode soil population densities, resulting in a 67% reduction in nematode densities relative to field plots planted with other commercially available soybean varieties. No differences in final reniform nematode soil population densities were observed between plots treated with AgLogic 15GG and non-treated plots. Differences in stand count were observed among certain varieties at each assessment date (Table 5). At 14 DAP, Bayer AG48XF2 and Dyna-Gro S48XT40 had significantly more plants per hectare than Progeny P4444RXS, University of Missouri S16-5503GT, University of Missouri S11-20195R, and WinField United Seed 48-D03. At 28 DAP, Bayer AG48XF2, Dyna-Gro S48XT40, Pioneer P46A86X, University of Missouri S16-5540R, University of Missouri S16-16641R, and WinField United Seed 46-D09 had a greater density of plants per hectare than Progeny P4444RXS, University of Missouri S16-5503GT, and University of Missouri S11-20195R. Application of AgLogic 15GG reduced plant establishment by 9.6% and 14.6% during the 14 DAP and 28 DAP measurements, respectively. Differences in yield were observed among tested varieties, with University of Missouri S16-5503GT, University of Missouri S16-16641R, and University of Missouri S11-2019R providing significantly higher yields than Bayer AG48XF2, Dyna-Gro S48XT40, Pioneer P46A86X, Progeny P4444RXS, Progeny P5252RX, University of Missouri S16-16814R, and WinField United Seed 46-D09. No differences in yield were observed between plots treated with AgLogic 15GG and plots without nematicide treatment.

**Table 4:** Influence of soybean variety and nematicide on *Rotylenchulus reniformis* soil population densities throughout the growing season at the MRRS field in 2022.

**Factor**	**Level**	***R. reniformis*/500 cm^3^ soil**		
		**At plant**	**Mid-season**	**At harvest**
Variety	Bayer AG48XF2	28,688 a	14,920 ab	4,824 bc
	Dyna-Gro S48XT40	20,192 a	12,712 a–c	3,248 bc
	Pioneer P46A86X	17,624 a	17,032 a	8,288 a–c
	Progeny P4444RXS	22,454 a	9,712 bc	13,032 a
	Progeny P5252RX	30,156 a	15,360 ab	10,440 ab
	University of Missouri S16-5503GT	41,457 a	11,240 bc	3,992 bc
	University of Missouri S16-5540R	25,283 a	10,344 bc	3,264 bc
	University of Missouri S16-16814R	25,606 a	8,648 c	3,464 bc
	University of Missouri S16-16641R	31,557 a	9,136 c	2,456 c
	University of Missouri S11-20195R	26,587 a	11,040 bc	2,200 c
	WinField United Seed 48-D03	21,256 a	18,352 a	12,992 a
	WinField United Seed 46-D09	19,100 a	10,608 bc	12,608 a

Nematicide	None	28,294 a	12,685 a	7,528 a
	AgLogic 15GG	23,366 a	12,165 a	5,940 a

*P*-values	Variety	0.084	0.009	0.005
	Nematicide	0.096	0.661	0.308
	Interaction	0.527	0.889	0.762

For each factor, values with common letters within a column do not differ significantly (*P*-value >0.05).

MRRS, Macon Ridge Research Station.

**Table 5:** Influence of soybean variety and nematicide on stand count and yield at the MRRS field in 2022.

**Factor**	**Level**	**Stand count (plants/ha)**		
		**14 DAP**	**28 DAP**	**Yield (kg/ha)**
Variety	Bayer AG48XF2	211,512 a	216,355 a–c	2,488 de
	Dyna-Gro S48XT40	213,449 a	221,522 a	2,825 c
	Pioneer P46A86X	190,199 ab	218,938 ab	2,287 e
	Progeny P4444RXS	173,730 c	171,147 e	2,421 e
	Progeny P5252RX	200,531 ab	190,520 de	2,488 de
	University of Missouri S16-5503GT	135,302 d	135,302 f	3,161 ab
	University of Missouri S16-5540R	198,272 ab	212,802 a–d	2,892 bc
	University of Missouri S16-16814R	198,272 ab	196,980 b–d	2,757 cd
	University of Missouri S16-16641R	209,251 ab	217,645 ab	3,295 a
	University of Missouri S11-20195R	136,595 d	125,616 f	3,161 ab
	WinField United Seed 48-D03	187,614 bc	192,781 c–e	2,959 bc
	WinField United Seed 46-D09	201,464 ab	209,896 a–d	2,825 c

Nematicide	None	197,464 a	207,635 a	2,825 a
	AgLogic 15GG	178,519 b	177,281 b	2,757 a

*P*-values	Variety	<0.001	<0.001	<0.001
	Nematicide	<0.001	<0.001	0.237
	Interaction	0.814	0.339	0.980

For each factor, values with common letters within a column do not differ significantly (*P*-value >0.05).

DAP, days after planting; MRRS, Macon Ridge Research Station.

### 2023 soybean growing season

At the NERS field in 2023, the reniform nematode soil population densities at the time of planting ranged between 18,368 and 34,496 nematodes per 500 cm^3^ of soil (Table 6). By mid-season, differences in reniform nematode soil population densities were observed. The field plots planted with the resistant University of Missouri soybean varieties had lower reniform nematode soil population densities relative to the field plots planted with other soybean varieties. At harvest, field plots planted with University of Missouri resistant soybean varieties continued to reduce reniform nematode soil population densities, resulting in 81% nematode reduction relative to field plots planted with the commercially available soybean varieties. At planting, root-knot nematode soil population densities averaged 0–768 nematodes per 500 cm^3^ of soil. No differences in root-knot nematode soil population densities were observed by mid-season or at harvest among varieties. Differences in stand count were observed among certain varieties at each assessment date (Table 7). At 14 DAP, Bayer AG48X9, Great Heart Seed GT-4677XS, University of Missouri S16-5503GT, University of Missouri S16-16814R, and University of Missouri S16-16641R had significantly more plants per hectare than Bayer AG48XF0 and Pioneer P54A54X. At 28 DAP, Bayer AG48X9, Great Heart Seed GT-4677XS, and University of Missouri S16-16814R had a greater density of plants per hectare than Bayer AG48XF0, Bayer AG48XF2, and Pioneer P54A54X. No differences in yield were observed among the tested varieties.

**Table 6:** Influence of soybean variety and nematicide on *Rotylenchulus reniformis* and *Meloidogyne incognita* soil population densities throughout the growing season at the NERS field in 2023.

**Variety**	***R. reniformis*/500 cm^3^ soil**			***M. incognita*/500 cm^3^ soil**		
	
	**At plant**	**Mid-season**	**At harvest**	**At plant**	**Mid-season**	**At harvest**
Bayer AG48XF0	18,368 a	16,640 a–d	24,704 ab	64 a	192 a	384 a
Bayer AG48XF2	19,008 a	28,160 a	26,624 ab	192 a	128 a	640 a
Bayer AG48X9	30,528 a	17,152 a–c	29,312 ab	128 a	0 a	192 a
Great Heart Seed GT-4677XS	25,136 a	22,272 ab	15,488 b–d	64 a	64 a	128 a
Local Seed Company LS5009XS	34,496 a	18,112 ab	30,400 a	768 a	192 a	1,728 a
Pioneer P54A54X	33,024 a	13,376 b–e	18,432 a–c	0 a	0 a	0 a
University of Missouri S16-5503GT	23,232 a	4,736 de	3,392 d	320 a	128 a	1,536 a
University of Missouri S16-5540R	28,352 a	5,952 c–e	7,936 cd	64 a	0 a	0 a
University of Missouri S16-16814R	20,416 a	2,752 e	3,456 d	64 a	0 a	256 a
University of Missouri S16-16641R	20,800 a	3,456 e	3,840 d	320 a	384 a	512 a

*P*-values	0.648	0.001	<0.001	0.436	0.413	0.584

Values with common letters within a column do not differ significantly (*P*-value >0.05).

NERS, Northeast Research Station.

**Table 7:** Influence of soybean variety on stand count and yield at the NERS field in 2023.

**Variety**	**Stand count (plants/ha)**		
	**14 DAP**	**28 DAP**	**Yield (kg/ha)**
Bayer AG48XF0	144,804 d	153,804 cd	3,901 a
Bayer AG48XF2	176,754 cd	142,083 cd	4,035 a
Bayer AG48X9	229,780 ab	190,350 ab	4,237 a
Great Heart Seed GT-4677XS	196,468 a–c	216,185 a	4,506 a
Local Seed Company LS5009XS	182,873 b–d	156,361 b–d	3,901 a
Pioneer P54A54X	135,965 d	129,167 d	4,842 a
University of Missouri S16-5503GT	204,628 a–c	161,117 b–d	4,775 a
University of Missouri S16-5540R	182,193 b–d	163,836 b–d	4,371 a
University of Missouri S16-16814R	239,978 a	212,105 a	4,304 a
University of Missouri S16-16641R	225,021 a–c	172,675 bc	4,842 a

*P*-values	0.001	<0.001	0.606

Values with common letters within a column do not differ significantly (*P*-value >0.05).

DAP, days after planting; NERS, Northeast Research Station.

At the MRRS field in 2023, reniform nematode soil population densities at the time of planting averaged 6,304–9,504 nematodes per 500 cm^3^ of soil (Table 8). No differences in reniform nematode soil population densities were observed by mid-season. At harvest, differences in reniform nematode soil population densities were observed among the varieties tested. Some commercial varieties, such as Bayer AG48XF2 and Bayer AG48X9, exhibited significantly higher soil population densities of the reniform nematode compared to the resistant University of Missouri varieties. Bayer AG48XF0 also displayed higher reniform nematode densities than University of Missouri S16-5540R and University of Missouri S16-16641R. Additionally, Great Heart Seed GT-4677XS showed greater reniform nematode population densities in the soil than University of Missouri S16-16641R. Differences in stand count were observed among certain varieties at each assessment date (Table 9). At 14 DAP, University of Missouri S16-16814R and University of Missouri S16-16641R had significantly more plants per hectare than Bayer AG48XF0, Bayer AG48XF2, Bayer AG48X9, and Local Seed Company LS5009XS. At 28 DAP, University of Missouri S16-16814R and University of Missouri S16-16641R had significantly more plants per hectare than all other varieties tested. Differences in yield were observed among tested varieties, with Local Seed Company LS5009XS, University of Missouri S16-5503GT, and University of Missouri S16-5540R providing significantly higher yields than Bayer AG48XF0, Pioneer P54A54X, University of Missouri S16-16814R, and University of Missouri S16-16641R.

**Table 8:** Influence of soybean variety on *Rotylenchulus reniformis* soil population densities throughout the growing season at the MRRS field in 2023.

**Variety**	***R. reniformis*/500 cm^3^ soil**		
	**At plant**	**Mid-season**	**At harvest**
Bayer AG48XF0	9,504 a	10,240 a	22,528 ab
Bayer AG48XF2	8,992 a	13,840 a	23,680 a
Bayer AG48X9	7,104 a	20,192 a	32,560 a
Great Heart Seed GT-4677XS	7,096 a	25,136 a	21,632 a–c
Local Seed Company LS5009XS	7,616 a	18,480 a	19,840 a–d
Pioneer P54A54X	6,304 a	16,128 a	20,720 a–d
University of Missouri S16-5503GT	8,800 a	10,592 a	8,960 b–c
University of Missouri S16-5540R	8,128 a	6,016 a	8,064 cd
University of Missouri S16-16814R	7,544 a	8,946 a	9,584 b–d
University of Missouri S16-16641R	9,376 a	8,512 a	7,684 d

*P*-values	0.998	0.115	0.007

Values with common letters within a column do not differ significantly (*P*-value >0.05).

MRRS, Macon Ridge Research Station.

**Table 9:** Influence of soybean variety on stand count and yield at the MRRS field in 2023.

**Variety**	**Stand count (plants/ha)**		
	**14 DAP**	**28 DAP**	**Yield (kg/ha)**
Bayer AG48XF0	146,163 c	131,887 c	1,614 cd
Bayer AG48XF2	121,010 c	127,808 c	1,950 b–d
Bayer AG48X9	153,640 c	151,601 c	1,883 b–d
Great Heart Seed GT-4677XS	161,117 bc	216,185 b	2,287 a–c
Local Seed Company LS5009XS	131,887 c	156,361 c	2,556 ab
Pioneer P54A54X	173,357 bc	162,479 bc	1,749 cd
University of Missouri S16-5503GT	189,670 a–c	178,113 bc	2,623 a
University of Missouri S16-5540R	173,357 bc	163,158 bc	2,556 ab
University of Missouri S16-16814R	256,295 a	293,005 a	1,480 d
University of Missouri S16-16641R	228,421 ab	281,448 a	1,749 cd

*P*-values	0.012	<0.001	0.013

Values with common letters within a column do not differ significantly (*P*-value > 0.05).

DAP, days after planting; MRRS, Macon Ridge Research Station.

## Discussion

In this study, reniform nematode-resistant soybean varieties consistently supported lower reniform nematode soil population densities at harvest compared to the commercially planted soybean varieties across both trial years (2022 and 2023) and field locations (NERS and MRRS). Averaged across both trial years, final reniform nematode soil population densities were 80% and 66% lower when planted with the resistant University of Missouri soybean varieties compared to the commercially planted soybean varieties at the NERS and MRRS fields, respectively. However, resistant University of Missouri soybean varieties did not reduce the population density of reniform nematodes in the soil at the MRRS during the 2023 trial. This lack in nematode suppression may be attributed to the presence of weeds (Davis and Webster, 2005), which may have facilitated nematode reproduction. Another possible explanation is that conducting the trial over two consecutive years may have allowed the nematode population to adapt and reproduce, thereby diminishing the effectiveness of the resistance trait. However, this is unlikely because we utilized different portions of the field between trial years. Overall, these findings highlight the need for additional research aimed at evaluating the role of weed hosts in supporting reniform nematode populations as well as the durability of deploying host resistance for reniform nematode management in soybean fields. Previous studies have shown that soybean varieties with resistance to reniform nematode are efficient in reducing the final soil population density of reniform nematode. For example, greenhouse studies by Robbins et al. (2001, 2002) revealed that some resistant varieties exhibited numerically smaller final nematode densities compared to the resistant check varieties ‘Forrest’ and ‘Hartwig’. Furthermore, reniform nematode-resistant soybean varieties have been successfully utilized in crop rotations to suppress reniform nematode soil population densities in certain areas of the cotton belt (Gilman et al., 1978; Williams et al., 1983; Davis et al., 2003). The present study and previous research demonstrate that reniform nematode-resistant soybean varieties may be an economically viable option for suppressing reniform nematode soil population densities in crop sequences that include highly susceptible crops, such as cotton and sweetpotato. In addition to suppressing reniform nematode population development in soil, many of the resistant soybean varieties had greater yields than the currently planted soybean varieties evaluated. Although greater yield cannot be directly attributed to reniform nematode suppression in the present study, given differences in agronomic traits associated with each soybean variety, suppression of reniform nematode feeding likely contributed to some extent toward greater yields, given the known susceptibility of soybean to reniform nematode in the region (McGawley et al., 2011). Findings from this study suggest that deploying reniform nematode host resistance in soybean can help reduce reniform nematode population development during the growing season, which may have other long-term benefits on subsequently planted susceptible rotation crops, including cotton and sweetpotato; however, this benefit still needs to be evaluated. In addition to the excellent suppression of reniform nematode population development observed in the present study, other studies have demonstrated the utility of some of these resistant soybean varieties evaluated for the suppression of other nematode pests and diseases. For example, University of Missouri S16-5503GT and University of Missouri S16-16641R have been evaluated in previous studies across various environments in the United States mid-southern states and demonstrated high-yield potential and resistance to multiple diseases such as stem canker and other nematodes of soybeans (Chen et al., 2023, 2024a, 2024b). Overall, the findings from the present study have demonstrated the utility of deploying reniform nematode host resistance in reniform nematode-infested soybean fields in Louisiana.

Despite the identification of reniform nematode resistance in previous studies, the underlying mechanisms of resistance in soybeans remain unclear. However, Rebois et al. (1970, 1975) pointed out that when the reniform nematode penetrates resistant soybean roots, a hypersensitive reaction typically occurs, resulting in cell death and ultimately inhibiting nematode development. The results of this study suggest that hypersensitive responses may be involved, given the complete lack of nematode population development observed in plots planted with resistant varieties. Consequently, this led to a significant suppression of reniform nematode soil population densities across both years of field trials. Furthermore, resistance has been confirmed in two distinct reniform nematode populations in this research. However, to verify the consistency of this resistance, further evaluation of multiple reniform nematode populations must be conducted. Thus, the present study provides valuable information for future studies on soybean resistance to the reniform nematode, including the identification of quantitative trait loci (QTL) associated with this resistance in soybeans. Such findings will ultimately be essential for future breeding programs aimed at developing soybean varieties with durable resistance to the reniform nematode.

In 2022, the in-furrow application of aldicarb (which is not registered for use on soybeans in Louisiana) was included as an additional factor in the experiment to evaluate the yield response of each soybean variety to the presence of nematodes. However, in both field locations, no discernible nematode suppression was observed due to the nematicide application. Similar results were obtained in a related study conducted in 2021 at the same field locations, where in-furrow-applied aldicarb did not affect reniform nematode population densities or the yield of 21 commonly planted commercial varieties (Watson and Price, 2022). Other studies have also demonstrated considerable variability in the efficacy of nematicides for suppressing reniform nematode (Koenning et al., 2007), including on soybeans (Lawrence et al., 2015). In addition to unstable performance, nematicides also have the potential to be toxic to human health, non-target organisms, and the environment (Desaeger et al., 2020). Their use necessitates strict handling practices and may be subject to increased regulatory restrictions or market withdrawal. The negative connotations associated with nematicides emphasize the importance of developing effective host resistance to manage the reniform nematode in soybean production. This study implies that deploying host resistance will be more beneficial for soybean growers compared to using nematicides. In addition, resistant soybean varieties may be incorporated into crop rotations with cotton and other susceptible crops to further reduce nematode soil population densities in infested fields. This rotational strategy may also minimize the selective pressure on reniform nematode populations to overcome host resistance, potentially preserving the durability of available resistant varieties. Overall, utilizing an integrated nematode management approach consisting of multiple management tactics will likely provide the most consistent level of nematode suppression and may help preserve the durability of host resistance in the future by preventing the development of resistance-breaking reniform nematode populations.

The present study has demonstrated that reniform nematode-resistant soybean varieties can effectively suppress reniform nematode population development in infested Louisiana fields and often result in greater yields than the currently grown commercial varieties. Further understanding of the mechanisms related to reniform nematode host resistance in soybeans and the genes involved in resistance will aid in the adoption of this trait into soybean breeding programs and may expedite the release of new resistant soybean varieties to this damaging pest. Furthermore, additional studies on the long-term benefit of deploying soybean host resistance on other reniform nematode-susceptible crops, including cotton and sweetpotato, are warranted.
